# Adsorption of Polystyrene from Theta Condition on Cellulose and Silica Studied by Quartz Crystal Microbalance

**DOI:** 10.1021/acs.langmuir.3c02777

**Published:** 2023-12-18

**Authors:** Katri S. Kontturi, Laleh Solhi, Eero Kontturi, Tekla Tammelin

**Affiliations:** † Biomass Processing and Products, VTT Technical Research Centre of Finland, FI-02044 Espoo, Finland; ‡ Department of Bioproducts and Biosystems, School of Chemical Engineering, Aalto University, P.O. Box 16300, 00076 Aalto, Finland

## Abstract

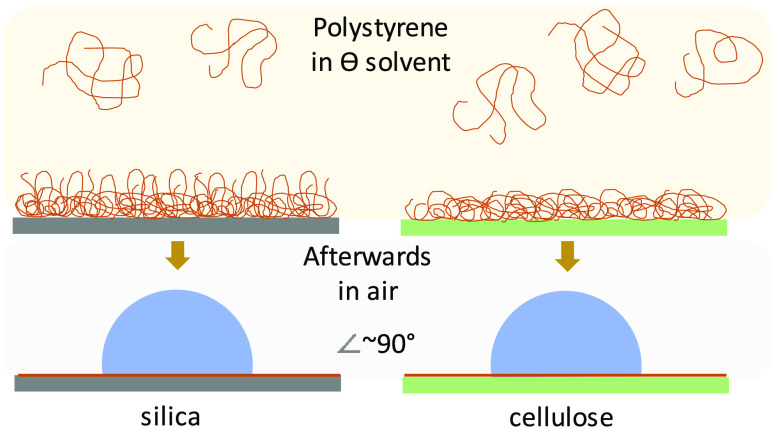

Adsorption of hydrophobic polymers from a nonpolar solvent medium is an underutilized tool for modification of surfaces, especially of soft matter. Adsorption of polystyrene (PS) from a theta solvent (50/50 vol % toluene/heptane) on ultrathin model films of cellulose was studied with a quartz crystal microbalance with dissipation monitoring (QCM-D), using three different PS grades with monodisperse molecular weights (*M*_w_s). Comparison of cellulose to silica as an adsorbent was presented. Adsorption on both surfaces was mainly irreversible under the studied conditions. Characteristically to polymer monolayer formation, the mass of the adsorbing polymer increased with its *M*_w_. The initial step of the layer formation was similar on both surfaces, but silica showed a stronger tendency for the formation of a loosely bound overlayer upon molecular rearrangements as the adsorption process proceeded. Despite the slightly less extended layers formed on cellulose at increasing *M*_w_ values, the overall thickness of the adsorbing wet layers on both surfaces was of the similar order of magnitude as the radius of gyration of the adsorbate molecule. Decent degree of hydrophobization of cellulose could be reached with all studied PS grades when the time allowed for adsorption was sufficient. QCM-D, a method conventionally utilized for studying aqueous systems, turned out to be a suitable tool for studying the adsorption process of hydrophobic polymers on soft polymeric matter such as cellulose taking place in a nonpolar solvent environment.

## Introduction

Ability to control and modify surfaces of any material is of crucial relevance because interfacial properties govern numerous application-related issues in the wide fields of emulsions and dispersions, composites, and coating technologies. One emerging area where alternative solutions for surface modifications are required is related to new uses of cellulose coupled with environmental demands, including replacing fossil-based materials in composite or packaging solutions as well as all functional high-end application possibilities enabled by nanocellulose.^1,2^

Polymer adsorption is an interesting, yet underutilized tool for modification of surfaces,^3,4^ the exception being polyelectrolyte adsorption, particularly in the form of a layer-by-layer deposition in an aqueous environment which is frequently used for adsorption-related modification of charged substrates, including biopolymers.^5^ However, adsorption of hydrophobic polymers in a nonpolar solvent medium is very rarely utilized in terms of surface modification. A sizable body of fundamental studies on hydrophobic polymer adsorption on inorganic substrates like metals or silica exist,^6−9^ but virtually no data on soft matter substrates—let alone biopolymer substrates—can be found. One exception is our recent proof-of-concept study concerning cellulose nanopaper modification with polystyrene (PS) and poly(trifluoro ethylene) (PTFE).^4^ It turned out that the relatively exotic PTFE had to be used in fairly high concentrations to impart reasonable hydrophobic character on the cellulose nanopaper substrate, while PS adsorption was generally not sufficient to have a meaningful impact on the surface properties of the nanopaper.

This study presents a fundamental effort to understand PS adsorption from a theta solvent (50/50 vol % toluene/heptane)^10^ on ultrathin model films of cellulose in a quartz crystal microbalance with dissipation monitoring (QCM-D). Theta solvent is a condition at the boundary between good and poor solvent quality where the polymer–polymer interaction is equally favorable with polymer–solvent interaction. In this condition, the polymer is molecularly dissolved in the solution adapting a random coil conformation with the size of the polymer coil directly proportional to its molecular weight (*M*_w_). Crucially, we could demonstrate that the adsorption kinetics of PS at this condition is relatively slow, efficient coverage reached within hours, indicating that a decent degree of hydrophobization of cellulose can be achieved by simple PS adsorption given that the time allowed for adsorption is sufficient. A systematic approach with three different PS grades with monodisperse *M*_w_s, coupled with the dynamic sensitivity of QCM-D, enables detailed charting of the adsorption phenomena. Moreover, we present a comparison of cellulose to a silica (SiO_2_) surface, which is one of the most common substrates in the literature on hydrophobic polymer adsorption. We also want to point out with this study that polymer adsorption on biobased surfaces does not need a specific chemical similarity between the adsorbent and the adsorbate to occur. By and large, polymers in solution adsorb on nearly any surface by entropic strive.^11^ It is often assumed in, for example, the community working with polysaccharides that “hydrogen bonding” or other specific interactions in one way or another must be involved for appreciable adsorption,^12^ but a detailed study on PS adsorption on a cellulose surface shows that this is certainly not the case. Although the demonstration here is set on hydrophobization of a cellulose surface, the concept is generic and basically applicable to any dissolving macromolecule, such as responsive or semiconducting polymers.

## Experimental Section

### Materials

Linear monodisperse (PDI 1.03–1.06) analytical grades of polystyrene with *M*_w_s of 10,000, 100,000, and 1,000,000 g/mol, denoted here as PS-10k, PS-100k, and PS-1M, respectively, were provided by Sigma-Aldrich. The properties of the polymers are presented in Table 1. Toluene (≥99.5%) and *n*-heptane (≥99.0%) of analytical grade were purchased from VWR Chemicals and used without further purification. Trimethylsilyl cellulose (TMSC) was synthesized from cellulose powder from spruce (Fluka) as described in ref (13). AT-cut quartz crystals with a fundamental resonance frequency *f*_0_ = 5 MHz and sensitivity constant *C* = 0.177 mg m^–2^ Hz^-1^ were obtained from Biolin Scientific (Gothenburg, Sweden).

**Table 1:** Properties of the Polymers

	*M*_w_ (g/mol)	polydispersity index (*M*_w_/*M_n_*)	radius of gyration (*R*_g_) in theta solvent^14^ (nm)
PS-10k	10^4^	1.06	2.70
PS-100k	10^5^	1.06	8.79
PS-1M	10^6^	1.03	28.56

### Preparation of the Polymer Solution

PS was dissolved in toluene by stirring at room temperature for 12 h. The solution was diluted with toluene to a concentration double the targeted final solution concentration, and *n*-heptane was added and mixed for a maximum of 5 min prior to injecting the solution to the QCM-D for the adsorption experiment. The prepared solutions contained 0.01, 0.1, 0.5, 1, and 2.5 g/L of PS (−10k, −100k, or −1M) in 50:50 vol % toluene/heptane solution. All PS solutions were stable and visually clear, with no cloudiness or precipitation. Molecular-level dissolution of the PS molecules in the solutions was also supported by the QCM-D response during the adsorption process (presented in the Results and Discussion Section), with Δ*f* and Δ*D* values at the range characteristic for adsorption of individual polymer chains reported for various polymer adsorption studies in the literature.^15−17^

Regarding the reproducibility of the measurements, it was found to be important to control the history of the polymer solution. For the stage of the equilibration process for the polymer molecule in theta solvent to be similar in all adsorption measurements, the antisolvent heptane was added to the PS-toluene solution only a few minutes before each adsorption experiment. In addition, the cleanliness of all glassware and equipment was maintained at the highest possible level throughout the experiments. The glassware cleansing protocol prior to use included (1) 48 h of soaking in a Deconex 11 UNIVERSAL detergent bath followed by rinsing with water, (2) laboratory dishwasher cleaning at 70 °C (45 min program) finishing by rinsing with distilled water, and (3) annealing at 250 °C overnight to ensure removal of any possible leftover organic contaminants.

### Preparation of the Surfaces

**Figure 1:**
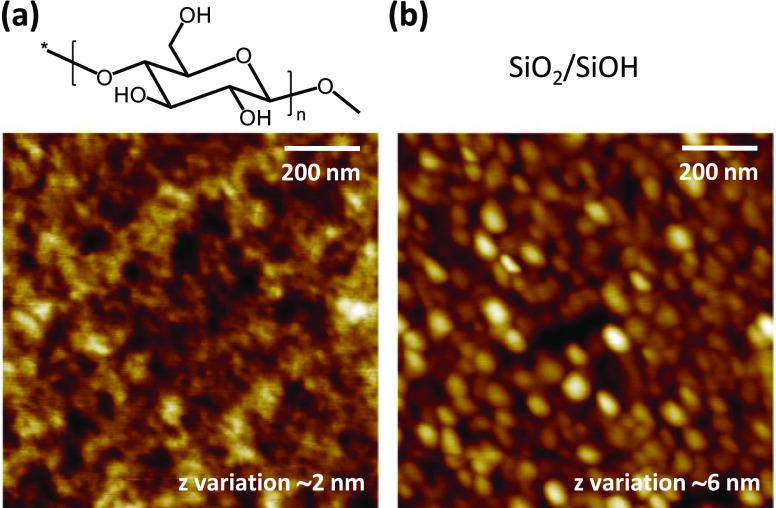
Chemical and morphological structure (AFM height images) of the (a) cellulose thin film and (b) silica layer deposited on the QCM-D sensor surface.

The cellulose surface was prepared by deposition of TMSC from 10 g/L toluene solution on a gold quartz crystal surface by spin coating at a spinning speed of 4000 rpm and subsequently hydrolyzing TMSC to cellulose by HCl vapor.^13^ Preparation of silica-coated sensors was performed via vapor deposition by the sensor manufacturer. Prior to the QCM-D experiment, the silica sensor surface was cleaned of any organic contaminants by ultraviolet (UV)/ozone treatment (Bioforce Nanosciences, Ames, Iowa) for 10 min. Atomic force microscopic (AFM) images of the cellulose and silica surfaces are presented in Figure 1.

### Characterization

Quartz crystal microbalance with dissipation monitoring (QCM-D) measurements were conducted using a Q-Sense E4 instrument (Biolin Scientific, Gothenburg, Sweden). In the QCM-D technique, a quartz crystal (coated with a thin layer of cellulose or silica in our case) is oscillated in pulses, and the changes of resonance frequency Δ*f* and dissipation of energy Δ*D* are measured as a function of time simultaneously at the fundamental frequency and its seven overtones. The mass adsorbed on the crystal surface is directly proportional to the detected Δ*f*, while the viscoelastic properties of the layer correlate with Δ*D*. The dissipation factor *D* of the oscillating adsorbing layer can be presented as
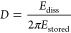
1where *E*_diss_ is the energy dissipated during an oscillation cycle and *E*_stored_ is the total energy stored in the oscillator. When the Δ*D* value for the adsorbed layer is relatively low, ≤1·10^–6^, the layer can be considered to be elastic, and it is acceptable to estimate the adsorbed dry mass using the Sauerbrey equation^18,19^
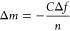
2where *C* is the sensitivity constant of the device (17.7 ng Hz^-1^ cm^–2^ for a 5 MHz crystal) and *n* is the overtone number.

In case the adsorbed layer does not meet the Sauerbrey conditions, the Voigt viscoelastic model^20^ (Dfind software, version 1.2.8, Biolin Scientific, Gothenburg, Sweden) was utilized for quantification of the wet layer areal mass and thickness. The parameters applied for modeling were density of 1.05 g cm^–3^ for PS, and density and viscosity of 0.77 g cm^–3^ and 0.46 mPa s for the toluene/heptane 50:50 Vol % mixture.

In this study, the quartz sensor surfaces were allowed to stabilize in a toluene/heptane 50:50 Vol % solution until a stable baseline was reached. During the adsorption measurement, the polymer solution was injected into the QCM-D cell as a continuous flow for 150 min. This was followed by rinsing with a pure solvent for 120 min. A temperature of 23.0 °C and a flow rate of 0.1 mL min^–1^ were maintained throughout the measurement. A minimum of two parallel measurements were recorded on each data point to ensure the repeatability and reliability of the data.

QCM-D is conventionally used for studying aqueous systems. Owing to the different density and viscosity of the toluene/heptane mixture compared to water, the absolute D values for QCM-D sensors measured in toluene/heptane were ∼34–39% lower than those characteristic for water. Thereby, some of the overtone signals were out of the range of “typical *D* values” indicated by the equipment manufacturer, which may have been realized as signal instabilities. Furthermore, especially the lower overtones tended to be disturbed by the occasional accumulation of air bubbles. Results of the normalized seventh overtone were more stable than the lower overtones and thus were selected for presenting Δ*f* and Δ*D* data. For the presentation of wet mass and thickness, viscoelastic modeling was utilized. Occasional artifacts caused by air bubbles were eliminated from the modeling by visual selection of a period of measurement time with the representative data points, enabling including the data of all overtones (3–11) for the modeling in most cases. Examples illustrating the appearance of the QCM-D raw data including typical artifacts are presented in Figure S1.

#### Viscosity and Density Measurement

Kinematic viscosities (ν) of the polymer solutions were determined using an Ubbelohde capillary viscometer immersed in a bath thermostatted to 23.0 °C. The Hagenbach and Couette correction factors were used to calculate the real efflux times. Five parallel measurements were performed on each sample. Densities (ρ) of the corresponding samples at 23.0 °C were determined by using a glass pycnometer. Subsequently, dynamic viscosity (η) of each sample was calculated by eq 3
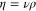
3

Atomic force microscopic (AFM) imaging was conducted using a Multimode 8 AFM instrument (Bruker AXS Inc., Madison, WI) to determine the morphology of the surfaces. The images were scanned in tapping mode in air at 25 °C using SiN cantilevers. No image processing except flattening was done.

Static contact angle measurements were conducted using an Attension Theta (Biolin Scientific, Gothenburg, Sweden) contact angle goniometer. The software delivered by the instrument manufacturer calculates the contact angles based on a numerical solution of the full Young–Laplace equation. The contact angle of water was measured on at least three locations on the QCM-D sensor surface after the QCM-D experiment and subsequent overnight drying. The original size of the water droplet was 2 μL.

The Cassie–Baxter equation^21^ was used for calculation of the surface coverage of PS on the cellulose and silica surfaces:
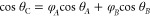
4where θ_C_ is the contact angle of the blend, ϕ_*A*_ and ϕ_*B*_ are the coverage of the components *A* and *B*, and θ_*A*_ and θ_*B*_ are the contact angles of the pure components.

## Results and Discussion

### Adsorption in Low Concentration

**Figure 2:**
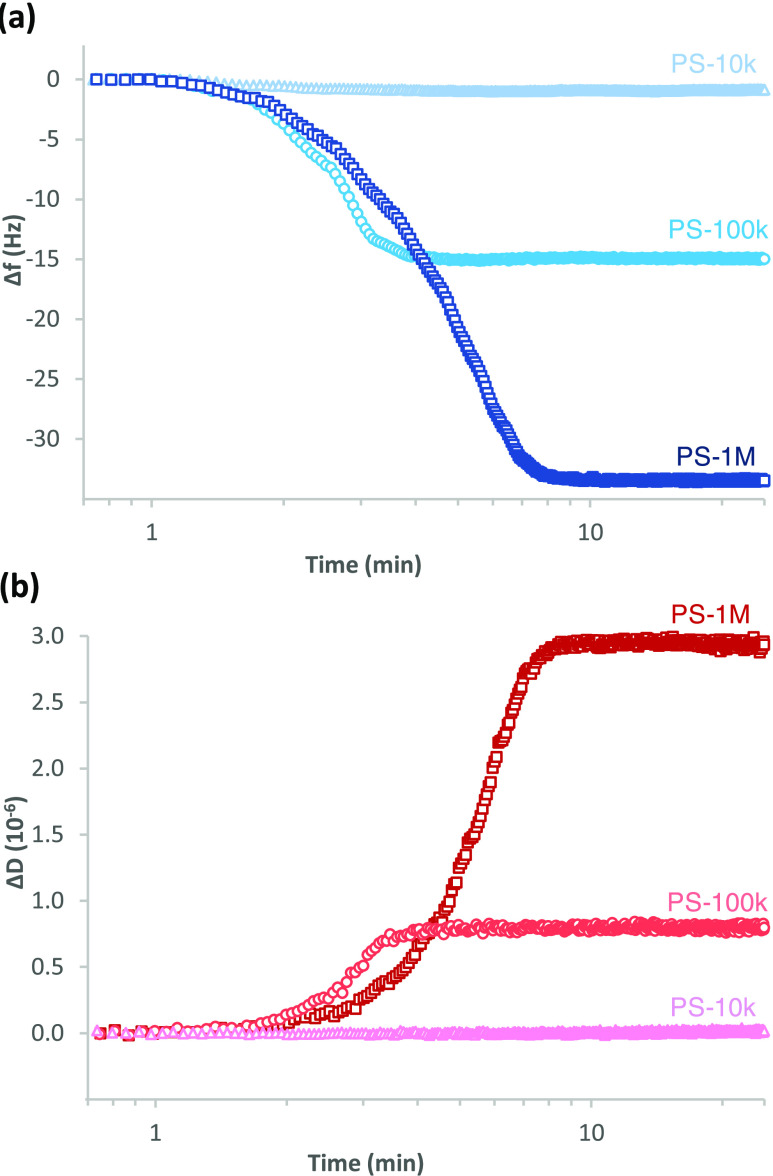
Changes in (a) frequency (Δ*f*) and (b) dissipation (Δ*D*) as a function of time during the initial stage of adsorption of 0.01 g/L PS-10k, PS-100k, and PS-1M from toluene/heptane on cellulose. The polymer is injected at *t* = 1 min, *f*_0_ = 5 MHz, *n* = 7, *f*_7_/*n*.

QCM-D response as a function of time during the initial stage of adsorption at a very low concentration of PS-10k, PS-100k, and PS-1M from toluene/heptane on cellulose is presented in Figure 2. The initial phase of polymer adsorption is limited by diffusion. In theta condition, the PS molecules appear in a random coil conformation, with *R*_g_ of the coil dependent on the *M*_w_ of the polymer as presented in Table 1. Larger molecular coils diffuse more slowly than the smaller ones, which explains the less steep overall slope for the change in frequency (Δ*f*) as well as the change in dissipation (Δ*D*) of PS-1M compared to those of the lower-*M*_w_ grades. The lowest-*M*_w_ PS-10k is able to diffuse and spread quickly on the surface, but the QCM-D response is quite weak due to the very small amount of adsorption: Δ*f* = −0.85 Hz and Δ*D* = 0.02·10^–6^ indicate the formation of a very dense and rigid layer with an areal mass of only 15.0 ng/cm^2^. The mass corresponds to a layer thickness of only 0.14 nm, indicating the adsorbed layer to be monomolecular (on an extended conformation) at maximum. The forming PS-100k layer (Δ*f* = −15.1 Hz and Δ*D* = 0.8·10^–6^) can also be considered rather rigid due to the relatively low (<1·10^–6^) Δ*D* value. The areal mass is 266.6 ng/cm^2^, corresponding to a (Sauerbrey) layer thickness of 2.51 nm. In the case of the PS-1M layer (Δ*f* = –33.5 Hz and Δ*D* = 2.9·10^–6^), on the other hand, the relatively high Δ*D* (≫1·10^–6^) value indicates the layer to be clearly softer compared to those of the lower-*M*_w_ PSs. The areal mass and thickness of the wet layer estimated based on viscoelastic modeling of QCM-D data are 900 ng/cm^2^ and 8.7 nm, respectively. An example of the fitting of the QCM-D data in the model is illustrated in Figure S1.

No notable difference was observed between the adsorption from low concentrations on cellulose and silica (Figure S2). For both surfaces, the Δ*f* and Δ*D* curves remain virtually unchanged after the initial stage, indicating no further detectable changes in the wet layer composition due to conformational rearrangement or further adsorption. The layer formation process appears typical for polymer adsorption at low concentration: in a diluted system, the number of macromolecules near the surface is low, and thus they can make a lot of contacts with the surface unhindered by other molecules. The polymer assumes a relatively flattened and dense conformation consisting of small loops or tails and a large number of train segments on the surface.

**Figure 3:**
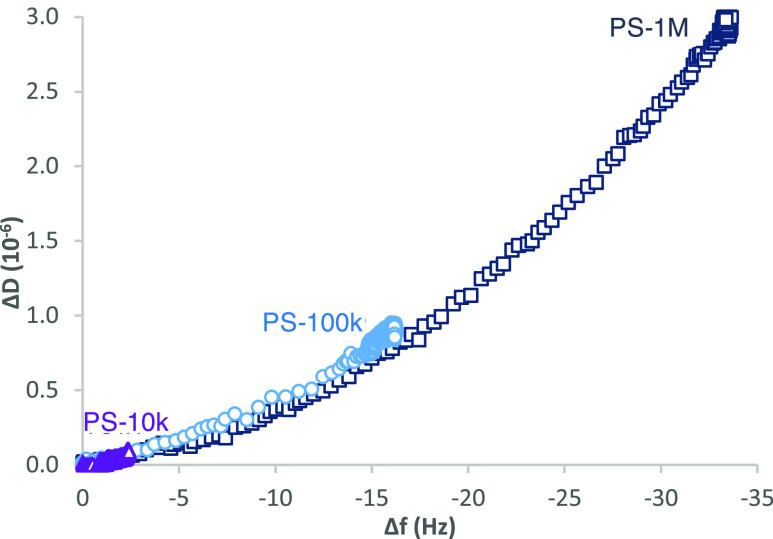
Change in dissipation (Δ*D*) as a function of change in frequency (Δ*f*) for the adsorption of 0.01 g/L PS-10k, PS-100k, and PS-1M from toluene/heptane on cellulose for 150 min, *f*_0_ = 5 MHz, *n* = 7, *f*_7_/*n*.

Value of Δ*D* as a function of Δ*f* for the adsorption of 0.01 g/L PS-10k, PS-100k, and PS-1M on cellulose is presented in Figure 3. This presentation eliminates time dependence from the QCM-D data and allows comparison of the layer development of different polymers. The overlapping curves indicate that the layer buildup follows the same pathway for different *M*_w_ samples—a certain mass increase corresponding to certain viscoelastic character of the (growing) layer. This indicates the formation of a homogeneous layer and is characteristic for a system where the interactions between the adsorbate and the adsorbent are relatively weak.

For comparison, in the case of polyelectrolyte adsorption onto an oppositely charged surface—a case of polymer adsorption where strong interactions are present—the forming polymer layer tends to become relatively thinner and laterally more heterogeneous than that in our case.^22^ During layer formation in such a system, the spacing between the adsorbing polymer chains is mainly restricted by the electrostatic repulsion between the molecules, while on the other hand, the electrostatic attraction toward the substrate results in flattened conformation of the readily adsorbed molecules. As a result, the development of Δ*D* vs Δ*f* for adsorption of polymer grades with constant charge density but varying sizes is not likely to overlap. This has been observed to be the case for adsorption of cationic starches on an anionic silica surface, where the higher-*M*_w_ polymer grade is not able to occupy the surface as efficiently as the lower-*M*_w_ grade due to conformational restrictions and repulsion between the adsorbing chains.^16^

Similar to the cellulose case, Δ*D* as a function of Δ*f* for the adsorption of different *M*_w_ PS grades on silica also follow an overlapping trend (Figure S3), indicating relatively unrestricted layer formation behavior. Thus, on both cellulose and silica surfaces, the interactions of styrene segments in each different-sized PS toward the adsorbent are similar. Furthermore, no detectable difference in the affinity of the PSs toward cellulose and silica can be observed at low concentration, where the competition of macromolecules for spare adsorption sites is expectedly negligible.

### Adsorption in Concentrated Solution

**Figure 4:**
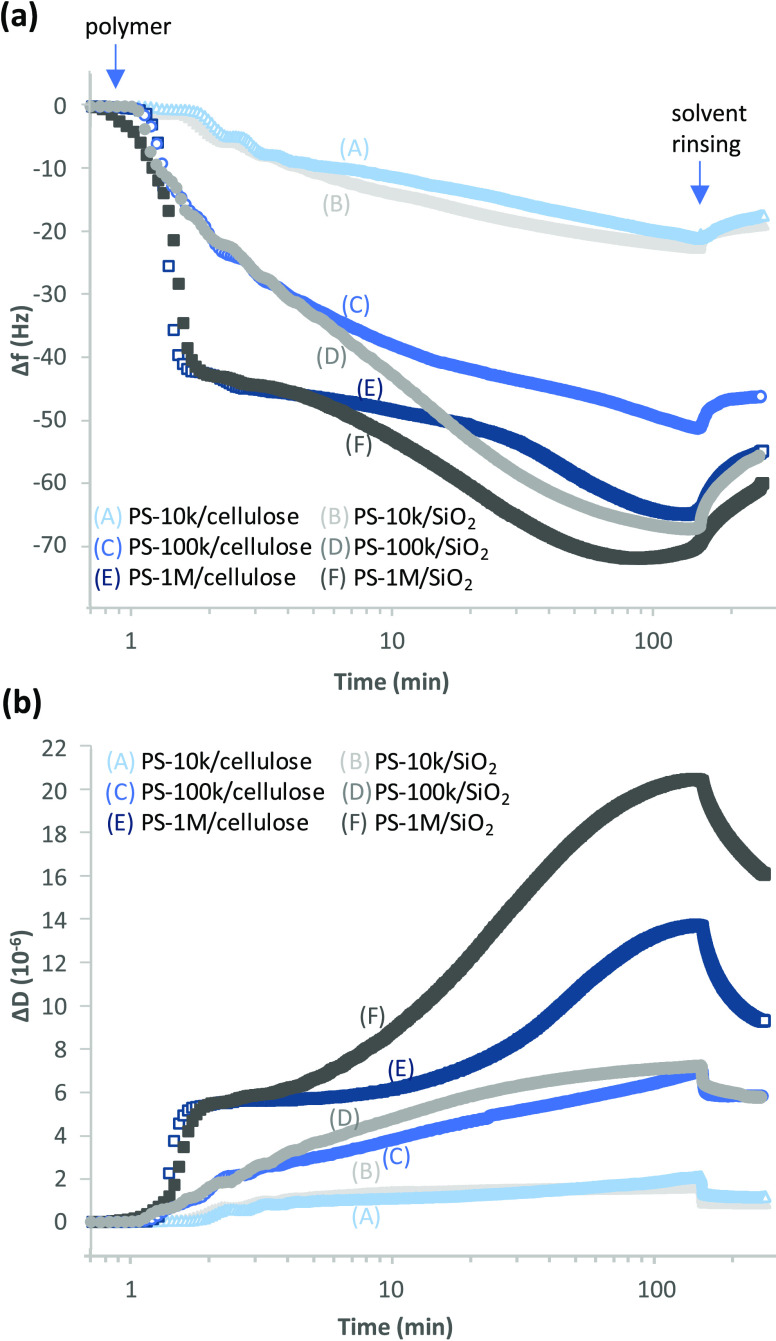
Changes in (a) frequency (Δ*f*) and (b) dissipation (Δ*D*) as a function of time during adsorption of 2.5 g/L PS-10k, PS-100k, and PS-1M from toluene/heptane on cellulose and silica. The injection of polymer at *t* = 1 min and the change to pure solvent at *t* = 150 min are indicated with arrows. The logarithmic time scale is selected to emphasize the kinetics of the early stages of adsorption. *f*_0_ = 5 MHz, *n* = 7, *f*_7_/*n*.

Changes in Δ*f* and Δ*D* as a function of time during adsorption of 2.5 g/L PS-10k, PS-100k, and PS-1M from toluene/heptane on cellulose and silica are presented in Figure 4. It is evident that the shapes of the time-dependent adsorption curves in this high concentration, close to the vertical initial stage followed by later curved phases changing with time, differ significantly compared to the obtuse angle shape followed by a constant horizontal line observed at low concentration (Figure 2).

The higher adsorption rate during the diffusion-limited initial phase, i.e., the steeper initial slope of Δ*f* and Δ*D* in Figure 4 compared to that of the low concentration situation in Figure 2, can be understood based simply on the concentration dependence of diffusion flux expressed by Fick’s law of diffusion.^23^ Regarding the interpretation of kinetics or adsorbed amounts during different stages of adsorption at high concentrations, it should be borne in mind that direct quantitative conclusions based on development of Δ*f* and Δ*D* are likely to be inaccurate due to the possible bulk effect. Bulk effect, Δ*f*_η_, is a deviation of the QCM-D signal caused by increase in viscosity and/or density of the solution compared to that of the pure solvent/buffer solution, according to eq 5:^24,25^
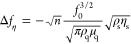
5where *f*_0_ is the fundamental resonance frequency of the quartz resonator under vacuum, ρ_q_ and μ_q_ are the density and the shear modulus of quartz; 2.6485 × 10^3^ kg m^–3^ and 2.92109 × 10^10^ N m^–2^,^25^ respectively, ρ_s_ and η_s_ are the density and dynamic viscosity of the solution, respectively, and *n* is the overtone number.

The values of  and the corresponding Δ*f*_η_ resolved with eq 5 for PS-10k, PS-100k, and PS-1M solutions in relevant concentrations are presented in Table 2. The values determined for ρ_s_ and η_s_ utilized in the calculations are presented in Table S1. The data indicate that the increased viscosities induce exaggeration of −2.8, −3.3, and −22.3 Hz in the recorded Δ*f* values for PS-10k, PS-100k, and PS-1M in high concentration, respectively. On the other hand, the minor difference between the values for the pure solvent and 0.01 g/L solutions confirms the bulk effect to be negligible in the low concentration QCM-D data (Figures 2 and 3). These kinds of bulk effect calculations might not necessarily provide numerically completely accurate treatment for the data^26^ but rather give an idea of the order of the magnitude for a possible viscosity-induced effect to be expected. Because of the possible bulk effect, the final numerical QCM-D values detected after the adsorption and subsequent period of rinsing with pure solvent provide more reliable and comparable data on the adsorbed mass and layer properties than the absolute values observed during the adsorption process.

**Table 2:** Values for the Square Root of the Product of Dynamic Viscosity and Density of the Solution, , and the Corresponding Bulk Effect, Δ*f*_*η*_, for PS-10k, PS-100k, and PS-1M in the Toluene/Heptane 50:50 Vol % Mixture at 23 °C in Different Concentrations

	PS-10k		PS-100k		PS-1M	
concentration (g/L)	a	Δ*f*_η_ (Hz)^b^	a	Δ*f*_η_ (Hz)^b^	a	Δ*f*_η_ (Hz)^b^
0	0.5929	0	0.5929	0	0.5929	0
0.01	0.5935	–0.18	0.5916	0.35	0.5932	–0.09
2.5	0.6032	–2.79	0.6052	–3.33	0.6751	–22.29

a Standard deviation: ±0.00066.

b Standard deviation: ±0.17 Hz.

Despite the limitations of interpretation of the absolute numerical values during the stages of adsorption at high concentrations with QCM-D due to the bulk effect, the shapes of the adsorption curves are informative. It is characteristic of polymer adsorption that competition of adsorbing macromolecules induces conformational rearrangements on the surface. Typically, during the diffusion-controlled first stage, all of the available surface sites are relatively quickly occupied by the adsorbing molecules without time for much arrangement. This is followed by the slower second stage, which typically involves rearrangement of the initially adsorbed macromolecules before any additional macromolecules can adsorb. While the early arriving macromolecules adopt a flatter conformation on the sparsely populated surface, the later arriving macromolecules have a smaller number of vacant sites available and, therefore, must adopt a much more extended conformation normal to the surface. As the solution bulk concentration increases, the competition for available sites increases, resulting in a decreased number of attachment sites per macromolecule and an increase in the thickness and typically a decrease in density of the wet adsorbed film.^6,27^

The steady gradual development of Δ*f* for adsorbing PS-10k and PS-100k layers indicates relatively efficient ability of the macromolecules to rearrange on both surfaces. Packing of the smallest molecule, PS-10k, is so efficient that the forming layers can be considered rigid (Δ*D* ∼ 1·10^–6^). On the other hand, with the increasing molecular size, the potential tail and loop length and thus the ability of the layer to bind solvent molecules also increases. The development of extensive tails over time is indicated by the significant increase of Δ*D* values of PS-1M.

As shown in the adsorption curves at low concentrations (Figures 2 and S2), the diffusion-controlled initial stage during the first minutes of adsorption on cellulose and silica is expectedly similar even at high concentrations (Figure 4). At high concentration, however, the Δ*f* and Δ*D* curves for cellulose and silica start to deviate from each other at 4–5 min due to conformational rearrangements, the wet PS layer developing on silica becoming heavier and looser than the corresponding layer on cellulose.

The rearrangement stage in a system of polymer adsorption at higher concentrations without specific attractive interactions might be very slow. Reaching an equilibrium during adsorption of PS on metal surfaces has been reported to take several days.^9^ Despite the much shorter adsorption (and subsequent desorption) times monitored in this study, the data provide us with an understanding of the reversibility and effect of the substrate on the adsorption process.

**Figure 5:**
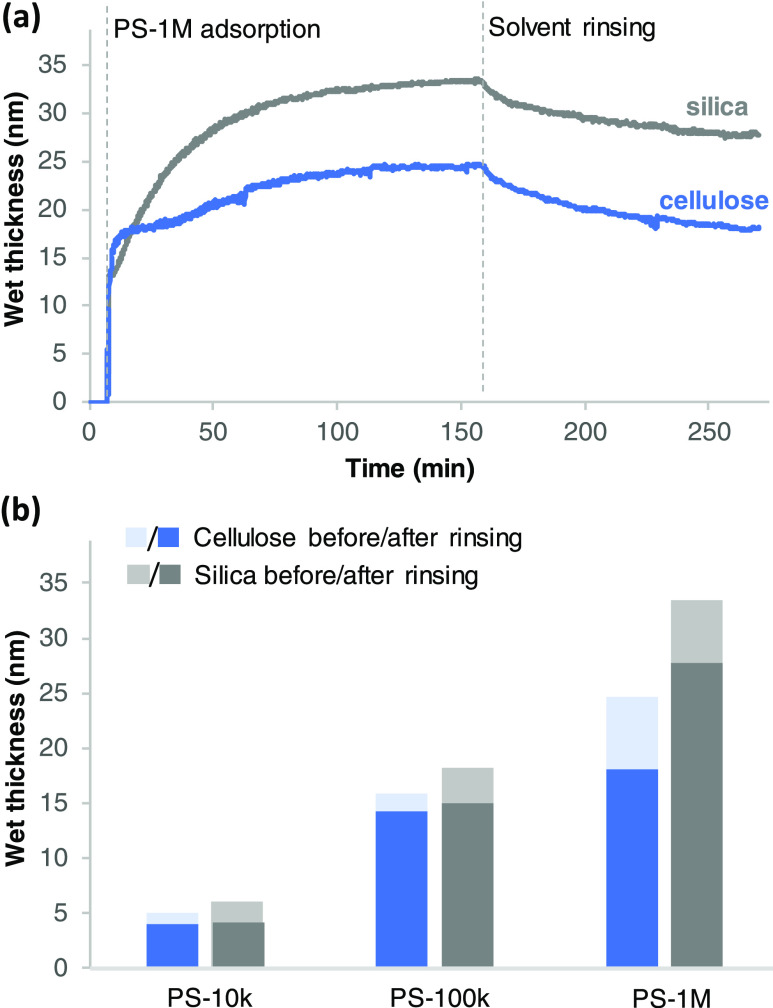
(a) Development of wet layer thickness on cellulose and silica as a function of time during adsorption of PS-1M from 2.5 g/L concentration and subsequent rinsing with pure solvent based on the modeling of the QCM-D data (fitting of the model into the measurement data presented in Figure S4), and (b) influence of solvent rinsing on wet thickness values of PS-10k, PS-100k, and PS-1M layers adsorbed on cellulose and silica from 2.5 g/L polymer concentration (“before” values correspond to the end of the adsorption phase at ∼160 min and “after” values the end of the subsequent solvent-rinsing phase at ∼270 min as in graph (a)).

Development of the PS-1M wet layer thickness during adsorption from concentrated solution and subsequent rinsing with pure solvent and the influence of solvent rinsing on wet thickness values of the different PS layers are presented in Figure 5. Adsorption on both cellulose and silica is mainly irreversible during the studied period of time. Some desorption takes place during rinsing of the adsorbed layer with pure solvent: the average decrease of thickness in all studied *M*_w_ values and concentrations was 12.1 ± 7.1% on cellulose and 15.9 ± 6.7% on silica. These desorption levels are in line with what has been reported for desorption of PS from theta solvent to polar inorganic surfaces from concentrated solutions.^28−31^ The development of wet layer thickness (Figure 5a) provides more insight regarding the stages of the layer formation process at concentrated solutions: the surface is instantly occupied by a layer of solvent-rich PS coils (resulting in a wet layer thickness of ∼13–17 nm for PS-1M), after which slow additional adsorption takes place over time as the rearrangements within the readily adsorbed layer proceed. Adsorption of the initially adsorbed macromolecules is irreversible on both surfaces, whereas the attachment of later-arriving macromolecules on cellulose is mainly reversible (thickness returning to the level of initial adsorption stage during rinsing) and on silica mainly irreversible (addition of 15 nm to wet layer thickness during the later stages of adsorption and subsequent rinsing). As shown in Figure 5b, the wet mass and thus the wet layer thickness adsorbed on silica significantly increase (becoming multiplied by ∼2–3 in all studied concentrations) with increasing magnitude of *M*_w_. Similar increasing trend of layer thickness with the magnitude of *M*_w_ has been observed for adsorption of PS on different metal surfaces with ellipsometry.^7^ On cellulose, the increase between PS-100k and PS-1M appears somewhat smaller.

### Adsorption Isotherms and Characteristics of the Layers of Maximum Coverage

**Figure 6:**
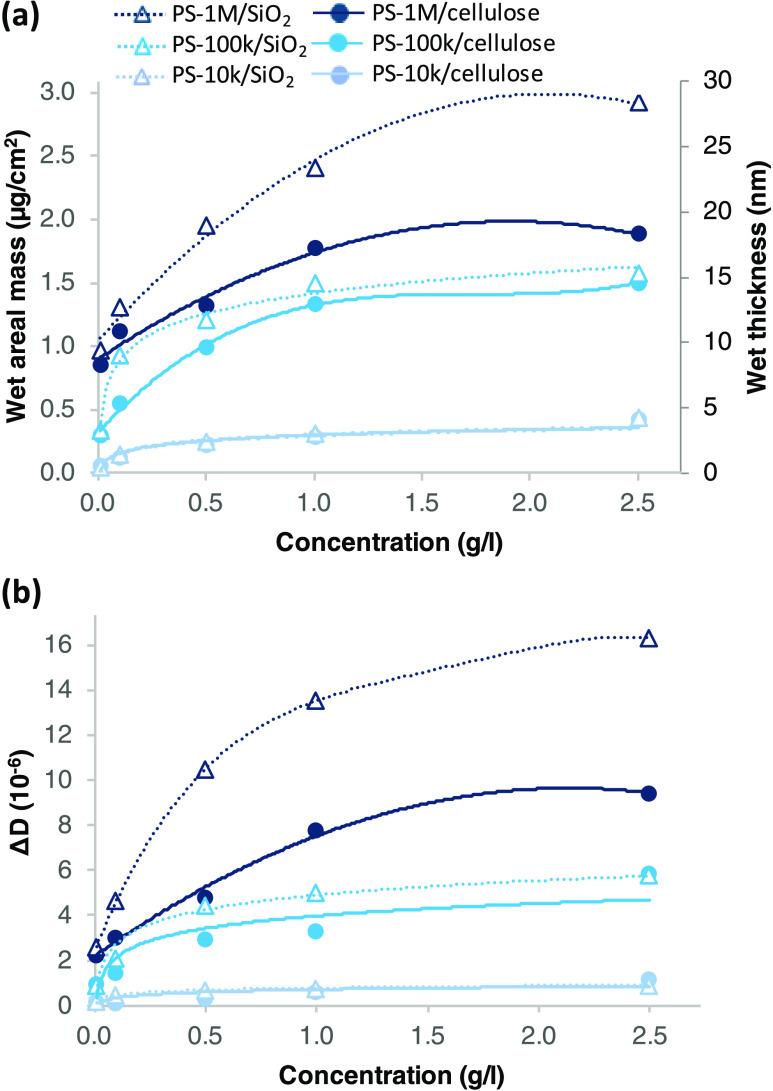
(a) Wet areal mass and wet layer thickness resolved by modeling of Δ*f* and Δ*D* data at available overtones (*n* = 3–11) and (b) change in dissipation (Δ*D*) at *n* = 7 due to irreversible adsorption of PS-10k, PS-100k, and PS-1M from toluene/heptane on cellulose and silica as a function of polymer concentration. The presented values are the result of an adsorption period (150 min) followed by a rinsing period (120 min). The lines are added to guide the eye. Note that the lines related to PS-10k on cellulose and silica in both graphs overlap.

The adsorption isotherms exhibiting irreversibly adsorbed wet areal mass and wet layer thickness of PSs in different concentrations as well as the corresponding dissipation Δ*D* values are presented in Figure 6. The wet adsorbed mass tends to increase with the bulk concentration of the solution until it levels off (Figure 6a). For PS-1M adsorbed on silica, however, the leveling-off concentration is not reached within the concentration range studied in this work. A similar trend was also observed by Killmann^7^ for adsorption of PSs on Cr from a theta solvent: The leveling-off concentration increases with *M*_w_ of PS, and for the highest *M*_w_ studied (750,000 g/mol in their work), the leveling off of the wet layer thickness was not reached yet even at 5 g/L.

According to the QCM-D data, the Δ*D* value of the layers (Figure 6b) also increases with both *M*_w_ and the solution concentration. The increase in viscous character is due to increased fractions of solvent-binding loops and tails within the adsorbed layer due to decreased fraction of the segments adsorbed per polymer chain.^6^ As a consequence, the dry adsorbed mass of a polymer also tends to increase with *M*_w_ and concentration until a plateau is reached. For the adsorption of PS on the silica surface, the dry mass adsorbed from theta solvent has been reported to increase with *M*_w_ of PS up to 500,000 g/mol and then level off.^27^

The layers adsorbed on silica generally possess higher wet masses and a higher dissipation of energy than those adsorbed on cellulose. This trend becomes pronounced as *M*_w_ increases.

Despite the slightly less extended layer of the high *M*_w_ PS formed on cellulose compared to that on silica, the wet thickness of the layers adsorbed on both surfaces are on the same order of magnitude with the *R*_g_ of different *M*_w_ PSs (Table 1). The layer thickness values on silica are on a similar level to those of different *M*_w_ PSs adsorbed on the Cr surface from another theta solvent cyclohexane at 36 °C measured with ellipsometry.^7^ Changing the adsorbent from Cr to Pt, or changing the theta solvent from cyclohexane to a THF/methanol mixture decreases the wet layer thickness by ∼25–50%. In the system with THF/methanol as a solvent, changing the absorbent from Cr to Au further decreases the wet layer thickness by ∼25–50%.^7^ Generally, the thickness values measured by ellipsometry might be somewhat underestimated to those compared to QCM-D: While QCM-D is able to detect the mass over the whole hydrodynamic thickness, ellipsometry only recognizes the layer where the refractive index is significantly different from the surrounding media, probably ignoring the long individual solvent-binding polymer tails likely protruding to the solution out of the layer. It is, however, interesting that the thicknesses observed on metal and silica surfaces are on a similar range despite the fact that the surface energies of the mentioned metal surfaces are close to 100-fold compared to that of silica (or cellulose).^32^

Thickness of the wet PS layer adsorbed on cellulose (∼7–12 nm) appears to be comparable or slightly higher than 6–10 nm thickness reported for carboxymethyl cellulose (CMC) adsorbed on regenerated cellulose in aqueous environment with increased ionic strengths (*M*_w_ 250,000 g/mol, polymer concentration 0.25 g/L, ionic strength 15–30 mM) based on a QCM-D study^17^ (comparable values on PS adsorption obtained by interpolation of *M*_w_ (between PS-100k and PS-1M) and concentration (between 0.1 and 0.5 g/L) in Figure 6a). CMC adsorption from high ionic strength can be seen as another case example of a coiled linear polymer with ring-structured repeating units adsorbing on a (regenerated) cellulose surface without specific attractive interaction, thereby serving as a fair reference for adsorption of PS. However, coiling of CMC at high ionic strength might be somewhat stronger than that of the random coil PS in theta solvent, possibly resulting in a relatively thinner wet adsorbed layer.

Similarly, in the case of the silica substrate, QCM-D adsorption studies resulting in wet layer thicknesses comparable to our PS layers can be found in the literature. The thickness of a layer of cationic starch (concentration 0.01 g/L) adsorbed on silica from aqueous solution with high ionic strength, where the polymer expectedly behaves like a neutral polymer, was 4.1 nm for a polymer with *M*_w_ of 450,000 g/mol and 7 nm for a polymer with *M*_w_ of 890,000 g/mol.^16^ These thickness values are in a similar range or slightly lower than those interpolated for the layers of the PS molecules of corresponding *M*_w_ values at 0.01 g/L (∼6–9 nm) based on Figure 6a. Similar to the adsorption of CMC on cellulose, cationic starch consists of ring-structured carbohydrate repeating units and adopts a somewhat coiled conformation in the solution due to the screening of intramolecular charges by the high ionic strength. In contrast to the linear CMC, however, it is likely that in cationic starch, there are also branched amylopectin fragments in addition to linear amylose present in the structure, potentially relatively decreasing the radius of the polymer coils compared to those of PS molecules of a corresponding *M*_w_.

**Figure 7:**
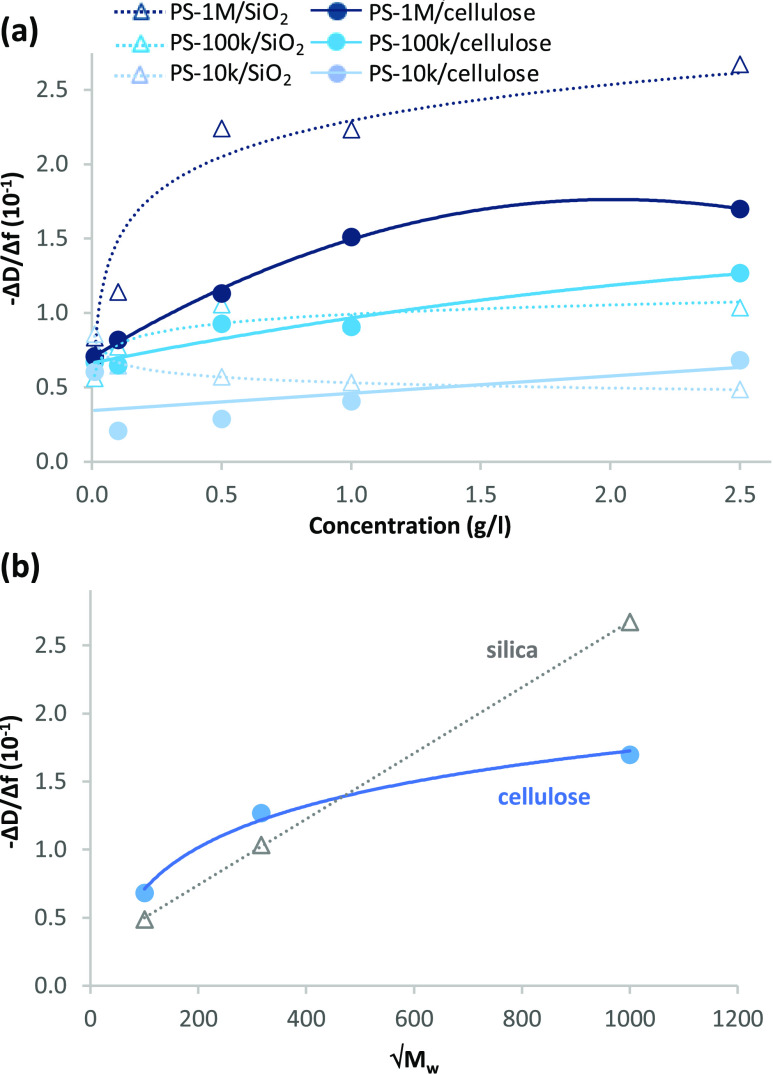
(a) Values of −Δ*D*/Δ*f* at *n* = 7 due to irreversible adsorption of PS-10k, PS-100k, and PS-1M from toluene/heptane on cellulose and silica as a function of polymer concentration, and (b) values of −Δ*D*/Δ*f* at 2.5 g/L concentration plotted as a function of square root of *M*_w_ of PS. The lines are added to guide the eye.

The adsorption isotherms of −Δ*D*/Δ*f* for irreversibly adsorbed wet layers of PSs and the corresponding values in a single concentration as a function of *√M*_w_ are presented in Figure 7. −Δ*D*/Δ*f* can be taken as a reference value for the relative proportion of viscosity to the elasticity of the adsorbed layer. Increase of the value with solution concentration for the larger polymers observed in PS-1M and PS-100k (Figure 7a) is characteristic for polymer adsorption since the number of solvent-binding loops and tails protruding out of the layer typically increases with bulk concentration in a case where no strong attraction between the polymer and the surface can be expected, as discussed earlier.^6^ No such clear increasing trend can be seen in PS-10k, probably due to its tight packing on the surface and thus the dominance of the elastic character over the viscous character in the layers (Δ*D* values < 1·10^–6^, Figure 6b). Of the more dissipative layers of PS-100k and PS-1M, −Δ*D*/Δ*f* values for PS-100k layers on cellulose and silica are relatively similar, indicating no dramatic differences between their viscoelastic properties. PS-1M layer, on the other hand, especially when adsorbed on silica, is remarkably soft and loose. Based on its −Δ*D*/Δ*f* value, the average viscoelastic characteristics of the PS-1M layers resemble that of a highly hygroscopic gel-like layer of CMC adsorbed on cellulose in the presence of increased ionic strength in aqueous environment, containing 90–95% water.^17^

**Figure 8:**
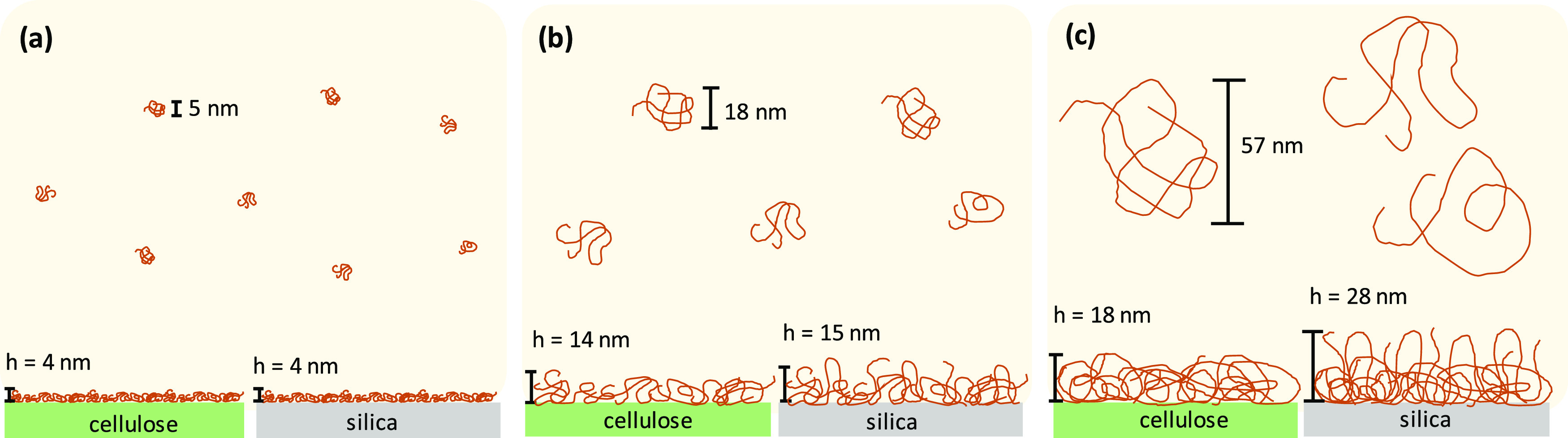
Scheme illustrating the correlation between the PS coil size in theta solvent and the wet thickness and appearance of the layer forming on the cellulose and silica surfaces upon adsorption from concentrated solutions for (a) PS-10k, (b) PS-100k, and (c) PS-1M.

The value of −Δ*D*/Δ*f* as a function of *√M*_w_ increases linearly on layers adsorbed on the silica substrate, as illustrated in Figure 7b. Similar linear correlation as a function of *√M*_w_ has been observed with ellipsometry for root-mean-square extension of wet PS layer adsorbed from a theta solvent on chrome and other metal surfaces.^7,9,33,34^ Like −Δ*D*/Δ*f*, also an increased rms extension due to increasing *M*_w_ of the polymer expectedly indicates decreased fraction of the adsorbing segments per macromolecule (adsorbed during the later stages of layer formation) and thus increased proportion of the solvent bound by the layer,^6^ which transpires as an increased viscosity/elasticity ratio. Since *R*_g_ of polymer molecules in theta solvent (Table 1) is also linearly dependent on *√M*_w_, it has been speculated that the linear correlation in the rms extension of the adsorbed layers on metal surfaces indicates adsorption of PS molecules on these surfaces to take place in random coil conformation.^7,33^ It seems reasonable to assume the adsorption of random coils, potentially with some degrees of coil flattening, to be the route for reaching instant coverage over the surface at the initial stage of adsorption in a case where strong attraction between the adsorbent and the adsorbate is lacking. The fact that the linear trend of −Δ*D*/Δ*f* as a function of *√M*_w_ in the current study applies only to silica while cellulose tends to bind high-*M*_w_ PS to a relatively denser layer (as indicated by the lower −Δ*D*/Δ*f* values at increasing *M*_w_s—the trend observed for all PS concentrations ≥0.1 g/L) indicates differences during the later stages of adsorption process between these surfaces. Such differences were also suggested by the difference in reversibility of the layer buildup (Figure 5a). After the initial stage, a loose overlayer is slowly forming as more molecules become capable of attaching to the surface upon rearrangements of the readily adsorbed molecules. The macromolecules in the overlayer have less contact points with the surface, and thus the density of this layer is low due to solvent-rich loops and tails extending to the solution. The overlayer formation is stronger on silica than cellulose surface. A scheme illustrating the appearance of PS coils in the solution and the corresponding PS layers after adsorption on the two surfaces is presented in Figure 8.

The fact that the overlayer formation is stronger on silica than on cellulose could be induced by a lower number of attachment sites on the cellulose surface compared to silica. It has been suggested in earlier studies that surface hydroxyl groups act as active surface sites in silica during adsorption of PS from nonpolar environments.^35,36^ Hydroxyl density of most silicas is 4–8 silanols/nm^2^,^37,38^ whereas the hydroxyl density of amorphous cellulose is approximately 2–4 OH/nm^2^.^39^ In our case, the surface area of the polycrystalline silica substrate is somewhat higher than that of cellulose due to its higher roughness (rms roughness values for cellulose and silica are 0.60 and 1.54 nm, respectively). The higher number of adsorption sites on silica enables irreversible attachment for macromolecules during the later stages of rearrangement and additional adsorption.

Despite the nonaqueous adsorption conditions, the presence of hydroxyls not only on cellulose but also on silica during the experiment is probably due to the fact that both cellulose and silica have a strong tendency to bind water molecules on their surfaces as soon as they are brought to ambient conditions. Avoiding short exposure to ambient conditions before subjecting the sensor surfaces to the solvent in the QCM-D flow cell would be practically impossible due to the limitations of the standard preparation procedure for QCM-D experiments.

From a thermodynamic point of view, the irreversibility of the buildup of additional loose overlayer after the initial adsorption step only on silica might also be a result of the higher polarity and (slightly) higher surface energy of silica; surface free energy and its polar component are 60.2 and 21 mN/m for cellulose, and 64.6 and 30.5 mN/m for silica, respectively.^40^ Higher surface energy against nonpolar environment induces stronger solvent molecule coordination and thus higher entropy gain upon the release of the solvent molecules from the interface during polymer adsorption. This promotes further adsorption, inducing the formation of an overlayer with extended loops and tails.

### Effect of Adsorbed Polymer Layers on Surface Properties

**Figure 9:**
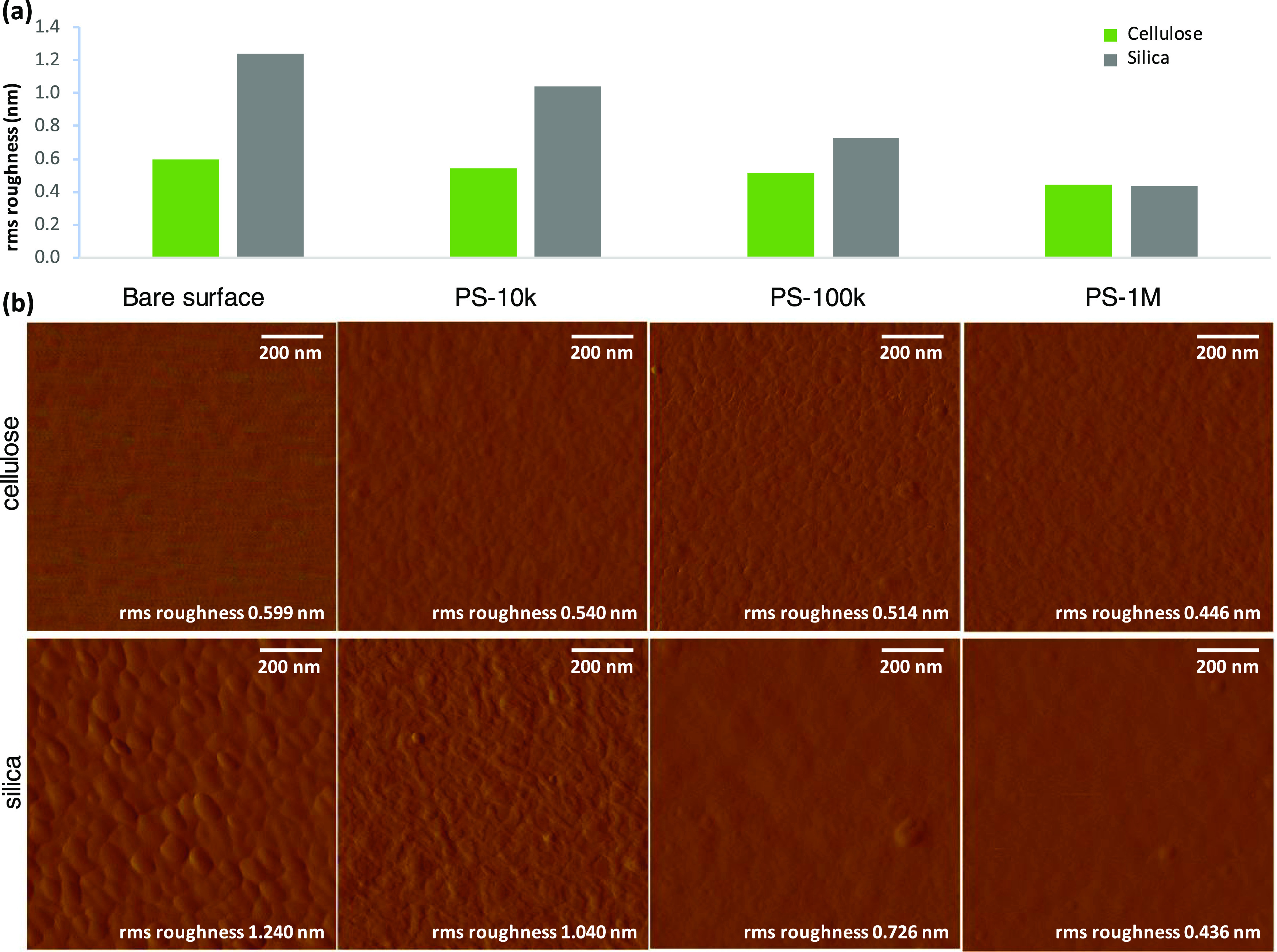
(a) Root-mean-square roughness of cellulose and silica surfaces after adsorption of PS-10k, PS-100k, and PS-1M from toluene/heptane from 2.5 g/L concentration, subsequent rinsing with pure solvent, and drying, and the corresponding (b) AFM 1 × 1 μm^2^ amplitude images (cellulose on top row and silica on bottom row).

As deduced from AFM images, the roughness of cellulose and silica surfaces after the polymer adsorption, subsequent rinsing with pure solvent, and drying is presented in Figure 9. It can be seen that the roughness of both surfaces decreases as a result of adsorption, the smoothening effect increasing with *M*_w_ of PS. The decreased roughness is an indication of increased dry mass of the adsorbed polymer layer as a function of *M*_w_, which is known to be characteristic for polymer adsorption.^6^ Adsorption of the highest-*M*_w_ PS-1M results in an equally low surface roughness on both substrates.

**Figure 10:**
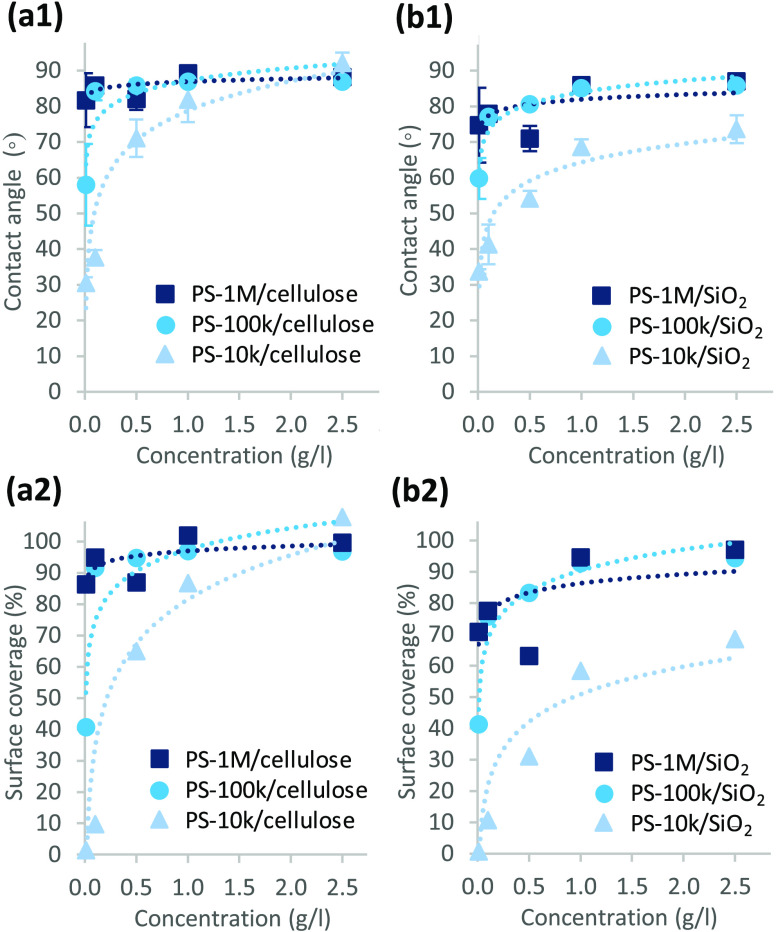
(a1, b1) Contact angle of water and (a2, b2) the corresponding coverage of the surface with polymer after adsorption of PS-10k, PS-100k, and PS-1M from toluene-heptane on (a) cellulose and (b) silica as a function of concentration. The surface treatment included the polymer adsorption step, rinsing with pure solvent, and subsequent drying. The surface coverage calculations are based on the Cassie–Baxter equation.

Contact angle of water on cellulose and silica surfaces after the adsorption experiment and subsequent drying, and the corresponding surface coverages based on the Cassie–Baxter equation^21^ (eq 4) are presented in Figure 10. The Cassie–Baxter calculation is a simple model expecting the effect of different components, in our case the polymer and the bare substrate, to have additive contributions for the wetting of the multicomponent surface. It is reasonable to assume that the wetting is affected merely by the chemical nature of the surface in our case; the roughness values of the studied samples are so low that they do not have noticeable influence on the contact angles.^41^ For all studied PSs, the contact angle increases with the concentration of the polymer solution used for adsorption, until a plateau close to the contact angle of pure polystyrene (88.42 ± 0.28°)^42^ is reached (Figure 10a,b). This indicates that the adsorbed dry mass follows a Langmuir-type isotherm. The higher the *M*_w_, the lower the polymer concentration required for the effective surface treatment, characteristically for polymer adsorption. This indicates that the maximum dry mass increases with *M*_w_, which is in agreement with the decreasing surface roughness as a function of *M*_w_ (Figure 9). The amount of PS-10k adsorbed from the plateau concentration is sufficient for the effective modification of cellulose, whereas the contact angle on silica after the corresponding treatment remains as low as 71.5°. Based on the Cassie–Baxter calculation of the additive components of the polymer and the surface, the surface coverage of PS-10k-treated silica is only ∼69%. This is likely an underestimated value since the calculation does not take into account the fact that not only the surface coverage but, in case of very thin films, also the very low PS layer thickness might decrease the contact angle; the contact angle has been found to decrease from 86 to 81° when the PS film thickness gradually decreased from 120 to 21 nm, the decrease getting more pronounced toward the lower values of the thickness range.^43^ Therefore, it is reasonable to assume that a thin layer with a full coverage of PS-10k has also formed on silica. For the same reason, since the adsorbed layer thicknesses are expectedly within the nanometer range, it is possible that other lower coverage values calculated based on the contact angle at lower polymer concentrations are also underestimated.

### Applicability of QCM-D for Monitoring Polymer Adsorption on Cellulose from Organic Solvent

No earlier record of literature on the adsorption process of hydrophobic polymers on soft polymeric matter such as cellulose taking place in a nonpolar solvent environment has been found. Based on the presented work, QCM-D turns out to be a suitable tool for studying such systems. The cellulose film showed no loss of mass or visible changes in morphology due to the exposure to the toluene/heptane 50:50 Vol % solvent mixture. Stabilization of the QCM-D sensors in the solvent took place rather quickly. No appreciable swelling or deswelling of the cellulose film was observed when subjected to the solvent; the average drift of the QCM-D signals for cellulose film under the solvent flowing 0.1 mL/min during 90 min (monitored for six samples) was Δ*f*_*n*_/*n* −0.3 ± 1.45 Hz and Δ*D*_*n*_/*n* 0.04 ± 0.07·10^–6^ (QCM-D data presented in Figure S5). This is in the acceptable range of any commercial inorganic QCM-D sensor surfaces; the maximum drift of the instrument as reported by the manufacturer is 2 Hz/h and 0.2·10^–6^/h at 15 MHz.^44^

Some instability issues for the QCM-D were caused by the unconventional solvent environment due to some overtone signals being out of range typical for aqueous systems, as well as a tendency for accumulation of air bubbles, as illustrated in Figure S1. Despite the occasional signal disturbances, the QCM-D data were generally reproducible and of decent quality, given that the meticulousness with the sample preparation procedures and cleanliness were maintained on a high level.

## Conclusions

Adsorption of PS on cellulose and silica from nonpolar theta solvent follows a Langmuir-type isotherm. Characteristically for polymer monolayer formation, the mass of the adsorbing polymer increases with its *M*_w_. Owing to a lack of specific attractive interactions, the adsorption process is slow. The time required for reaching effective surface coverage increases with *M*_w_ of the polymer and the concentration of the solution. ∼2 h adsorption time is adequate for building a layer of full coverage of PS with M_w_ of 1,000,000 g/mol adsorbed from 2.5 g/L solution on either cellulose or silica. The adsorption is mainly irreversible under the studied period of time.

The initial step of the layer formation process on cellulose and silica is similar, but additional slow attachment of polymer molecules on the surface upon rearrangements of the readily adsorbed macromolecules, resulting in buildup of a solvent-rich overlayer, is stronger on silica than on cellulose. The more significant overlayer formation on silica is likely due to the relatively larger number of adsorption sites and higher polarity of silica compared with those of cellulose. Generally, the dependency between the √*M*_w_ of the adsorbate molecule and the overall viscoelastic characteristics of the corresponding wet layer adsorbed on silica follows a similar linear trend that has been reported in the literature for metal surfaces. Despite the somewhat less extended layers formed on cellulose than on silica at higher *M*_w_ values, the wet thickness of the layers adsorbed at high concentrations on both surfaces is of a similar order of magnitude as the radius of gyration of the adsorbate molecule in the solution. Especially at high *M*_w_, such a layer has viscous, gel-like consistency in the wet state. When the solvent is excluded from the system during drying after the adsorption treatment, the adsorbed polymer layer densifies into an effective coating on both cellulose and silica.

Polymer adsorption from nonpolar (theta) solvents on polar surfaces opens wide possibilities for straightforward modification of soft matter, such as cellulose and other material surfaces. The fact that nonpolar solvents do not swell or disperse cellulose as water does makes the approach applicable for modification of either individual (nano)fibers or readily built macroscopic architectures. QCM-D proved to be an applicable method for studying the layer formation process in such systems. Knowing the characteristics of the wet adsorbing layer and the conformities during the film formation process enables tuning the targeted coverage and coating properties by selection of optimal *M*_w_, concentration, solvent, and process time. On the other hand, potential highly dissipative adsorption stages could be utilized for encapsulation of additional components such as cross-linkers or nanoparticle doping within the layer to build composite layers or other functionalized coatings.

## Supporting Information Available

QCM-D raw data to illustrate examples of fitting of viscoelastic model and occasional measurement artifacts; QCM-D data of adsorption of PS grades from low concentration on silica; QCM-D stabilization data of cellulose-coated sensor in toluene/heptane; and kinematic viscosities and densities of the polymer solutions (PDF)
