# Supplementary material for: Adsorption of Polystyrene from Theta Condition on Cellulose and Silica Studied by Quartz Crystal Microbalance
Source: Langmuir. 2023 Dec 18;40(1):568–79. doi: 10.1021/acs.langmuir.3c02777 (PMC10786068; doi:10.1021/acs.langmuir.3c02777)
Supplement: Supplementary file 1 — la3c02777_si_001.pdf [file la3c02777_si_001.pdf]

Supporting information

for

Adsorption of polystyrene from theta condition on cellulose and silica studied by quartz crystal microbalance

by

Katri S. Kontturi,[a*] Laleh Solhi,[b] Eero Kontturi,[b] Tekla Tammelin[a]

a) Biomass Processing and Products, VTT Technical Research Centre of Finland, FI-02044 Espoo, Finland

b) Department of Bioproducts and Biosystems, School of Chemical Engineering, Aalto University, 00076 Aalto, Finland

*) corresponding author, contact: katri.kontturi@vtt.fi

TABLE OF CONTENTS

Figure S1. Examples of QCM-D raw data for two systems with occasional artefacts, and corresponding best fit of Voigt-based viscoelastic model to the data.

Figure S2. Changes in frequency and dissipation during the initial stage of adsorption of 0.01 g/l PS-10k, PS-100k, and PS-1M on silica.

Figure S3. Change in dissipation as a function of change in frequency for the adsorption of 0.01 g/l PS-10k, PS-100k, and PS-1M on silica for 150 min.

Figure S4. QCM-D raw data for adsorption of 2.5 g/l PS-1M on cellulose and silica, and corresponding best fit of Voigt-based viscoelastic model to the data.

Figure S5. Development of Δf and ΔD during stabilization period of cellulose-coated QCM-D sensors.

Table S1. Kinematic viscosity and density of polymer solutions as defined with capillary viscometry and pycnometry, and the dynamic viscosity calculated thereof.

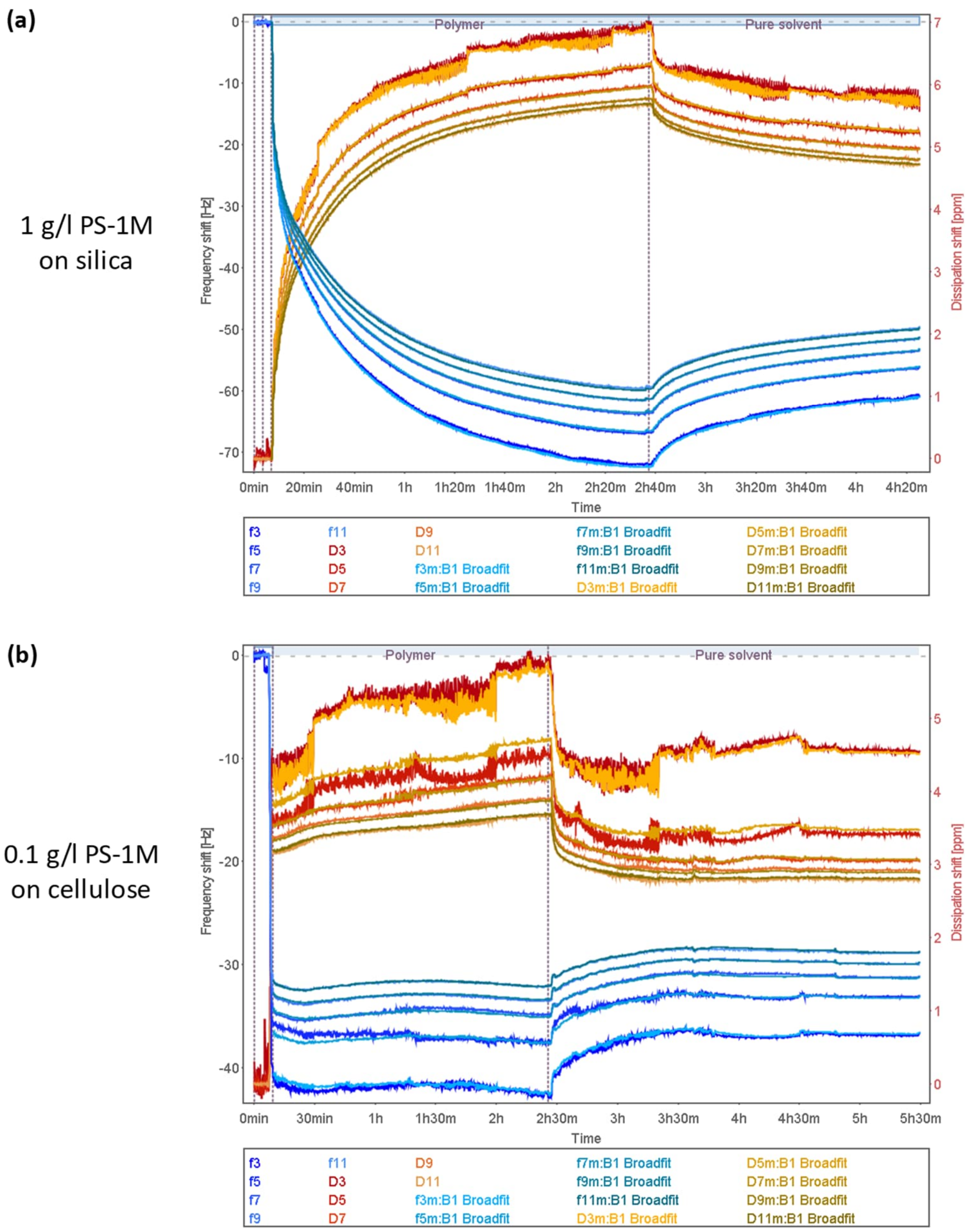

Figure S1. Examples of QCM-D raw data monitored at 15, 25, 35, 45, and 55 MHz (n = 3, 5, 7, 9, and 11; frequencies (f) in blue and dissipation values (D) in red) as a function of time for a system with (a) moderate and (b) severe occasional disturbance caused by air bubbles, and corresponding best fit of Voigt-based viscoelastic model (Voinova et al. 1999) to the data by Dfind software (fitted values for f in turquoise and for D in yellow). For the modeling, the density for polystyrene was assumed as 1.05 g cm^{-3}, and density and viscosity for toluene-heptane 50:50 Vol-% mixture as 0.77 g cm^{-3} and 0.46 mPa s.

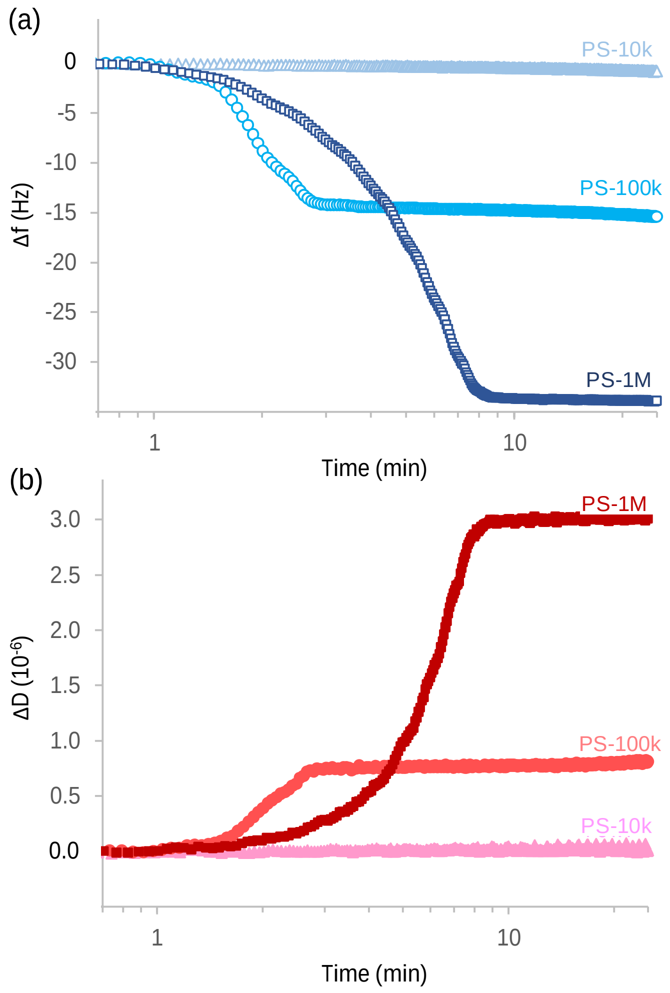

Figure S2. Changes in (a) frequency (Δf) and (b) dissipation (ΔD) as a function of time during the initial stage of adsorption of 0.01 g/l PS-10k, PS-100k, and PS-1M from 50:50 Vol-% toluene/heptane on silica (n=7). The polymer is injected at t = 1 min.

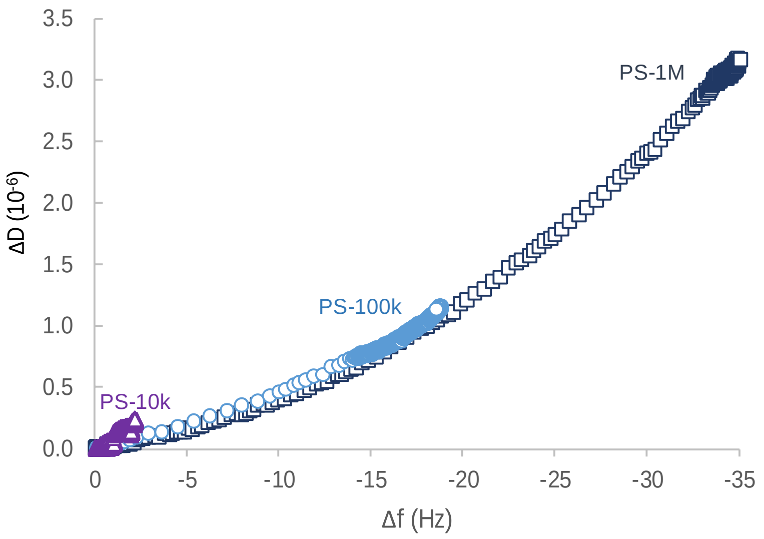

Figure S3. Change in dissipation (Δ*D*) as a function of change in frequency (Δ*f*) for the adsorption of 0.01 g/l PS-10k, PS-100k, and PS-1M from toluene/heptane on silica for 150 min (*n*=7). Some interference caused by air bubbles occurred in the signal of PS k4 adsorption.

(a)

2.5 g/l PS-1M on cellulose

(b)

2.5 g/l PS-1M on silica

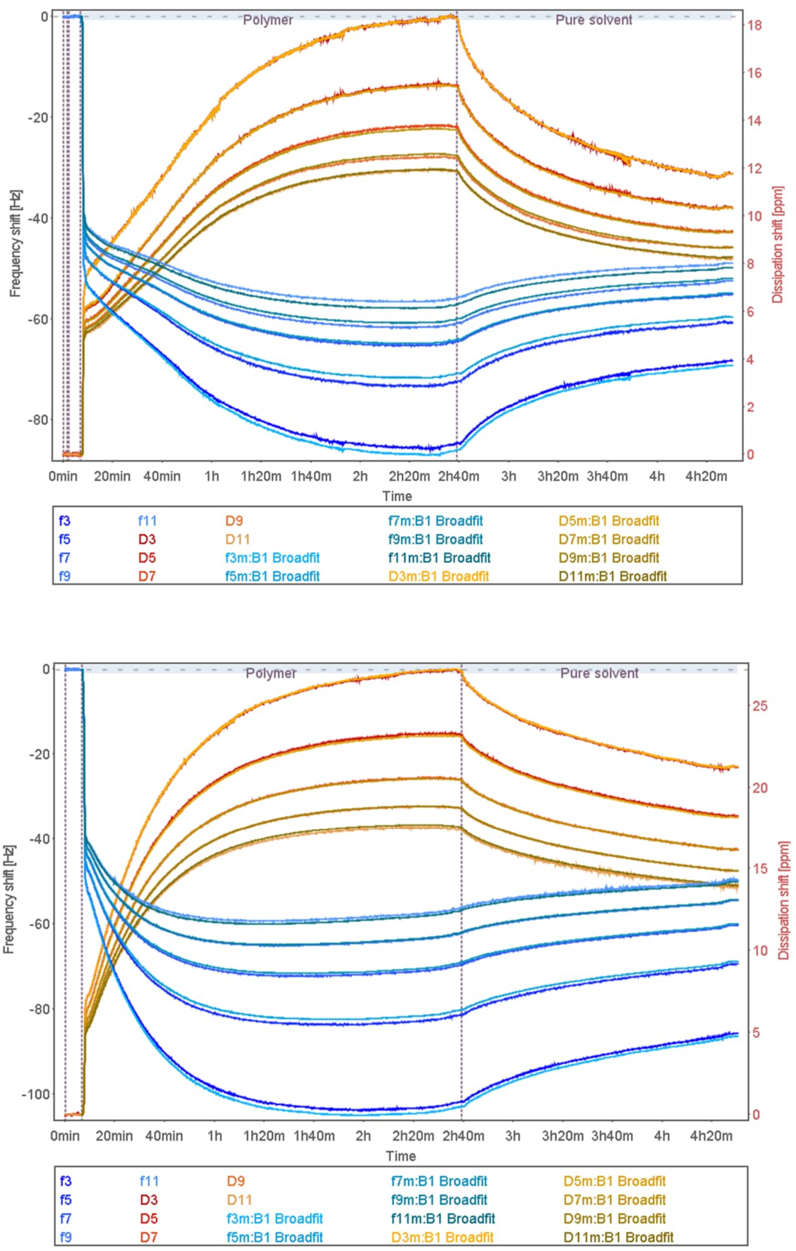

Figure S4. QCM-D raw data monitored at 15, 25, 35, 45, and 55 MHz (n = 3, 5, 7, 9, and 11; frequencies (f) in blue and dissipation values (D) in red) as a function of time for adsorption of 2.5 g/l PS-1M on (a) cellulose and (b) silica, and corresponding best fit of Voigt-based viscoelastic model (Voinova et al. 1999) to the data by Dfind software (fitted values for f in turquoise and for D in yellow). For the modeling, the density for polystyrene was assumed as 1.05 g cm^{-3}, and density and viscosity for toluene-heptane 50:50 Vol-% mixture as 0.77 g cm^{-3} and 0.46 mPa s.

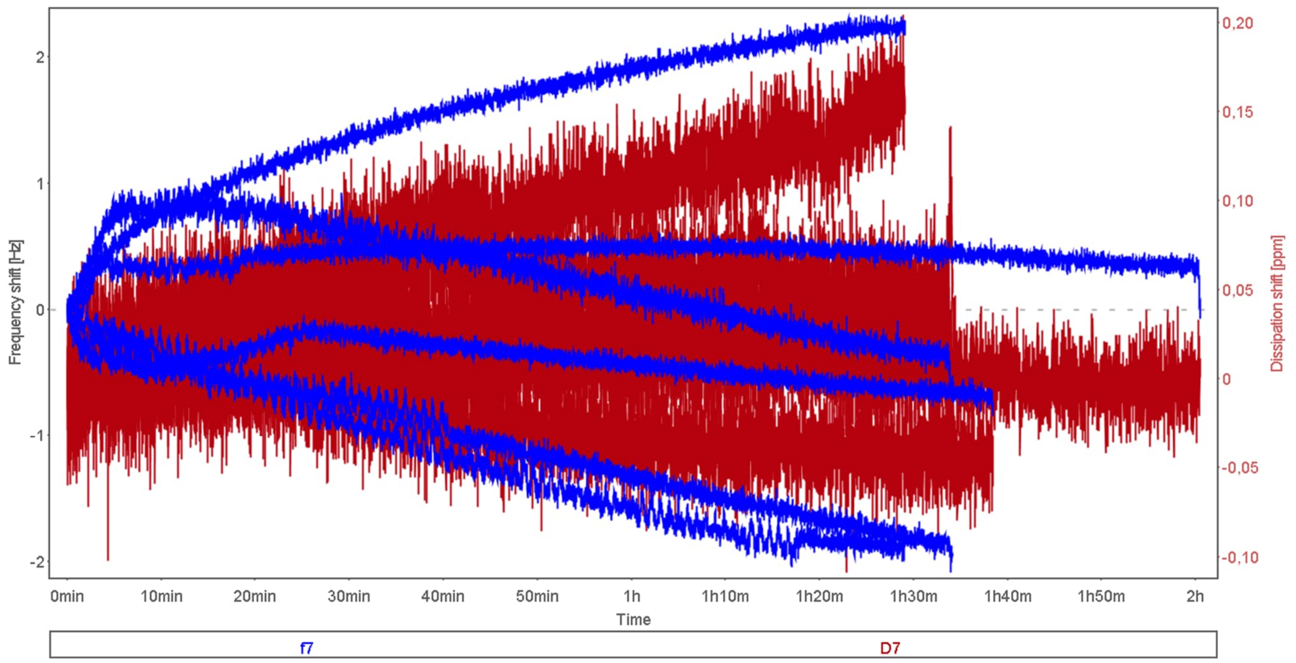

Figure S5. Development of Δf (blue) and ΔD (red) (n = 7) during stabilisation period prior to adsorption measurements of six parallel cellulose-coated QCM-D sensors in toluene/heptane 50:50 Vol-% for 90 minutes.

Table S1. Kinematic viscosity and density of polymer solutions as defined with capillary viscometry and pycnometry, and the dynamic viscosity calculated thereof.

	Kinematic viscosity* ν_s (10^{-6} m^2/s)			Density, ρ_s (g/cm^3)			Dynamic viscosity, $\eta_s = \nu_s \times \rho_s$ (mPa s)		
Conc.	PS-10k	PS-100k	PS-1M	PS-10k	PS-100k	PS-1M	PS-10k	PS-100k	PS-1M
0 g/l	0.59314	0.59314	0.59314	0.76980	0.76980	0.76980	0.45660	0.45660	0.45660
0.01 g/l	0.59337	0.59287	0.59423	0.77049	0.76827	0.76953	0.45719	0.45549	0.45727
0.1 g/l	0.59350	0.59318	0.60066	0.77034	0.76864	0.76926	0.45719	0.45595	0.46206
0.5 g/l	0.59348	0.59932	0.62592	0.76999	0.76984	0.77005	0.45697	0.46138	0.48199
1 g/l	0.59822	0.60247	0.65548	0.76984	0.76970	0.77067	0.46053	0.46372	0.50515
2.5 g/l	0.60817	0.61393	0.76436	0.77342	0.77234	0.77217	0.47037	0.47417	0.59022

*) standard deviation ±0.5%
